# Highly sensitive detection of the group A Rotavirus using Apolipoprotein H-coated ELISA plates compared to quantitative real-time PCR

**DOI:** 10.1186/1743-422X-8-63

**Published:** 2011-02-10

**Authors:** Cornelia Adlhoch, Marco Kaiser, Marina Hoehne, Andreas Mas Marques, Ilias Stefas, Francisco Veas, Heinz Ellerbrok

**Affiliations:** 1 Robert Koch Institute, Center for Biological Security ZBS1, Nordufer 20, 13353 Berlin, Germany; 2 GenExpress, Eresburgstr. 22-23, 12103 Berlin, Germany; 3 Robert Koch Institute, Division of Molecular Epidemiology of Viral Pathogens, Nordufer 20, 13353 Berlin, Germany; 4 ApoH Technologies, University Montpellier-1, Faculty of Pharmacy, 15 Ave. Charles Flahault, Building D, 34093 Montpellier, France; 5 Institut de Recherche pour le Développement, University Montpellier-1, Viral and Molecular Immunology Laboratory, U178/IRD Emerging Diseases, Faculty of Pharmacy, 15 Ave. Charles Flahault, Building D, 34093 Montpellier, France

## Abstract

**Background:**

The principle of a capture ELISA is binding of specific capture antibodies (polyclonal or monoclonal) to the surface of a suitable 96 well plate. These immobilized antibodies are capable of specifically binding a virus present in a clinical sample. Subsequently, the captured virus is detected using a specific detection antibody. The drawback of this method is that a capture ELISA can only function for a single virus captured by the primary antibody. Human Apolipoprotein H (ApoH) or β_2_-glycoprotein 1 is able to poly-specifically bind viral pathogens. Replacing specific capture antibodies by ApoH should allow poly-specific capture of different viruses that subsequently could be revealed using specific detection antibodies. Thus, using a single capture ELISA format different viruses could be analysed depending on the detection antibody that is applied. In order to demonstrate that this is a valid approach we show detection of group A rotaviruses from stool samples as a proof of principle for a new method of capture ELISA that should also be applicable to other viruses.

**Results:**

Stool samples of different circulating common human and potentially zoonotic group A rotavirus strains, which were pretested in commercial EIAs and genotyped by PCR, were tested in parallel in an ApoH-ELISA set-up and by quantitative real-time PCR (qPCR). Several control samples were included in the analysis. The ApoH-ELISA was suitable for the capture of rotavirus-particles and the detection down to 1,000 infectious units (TCID_50/ml_). Subsets of diagnostic samples of different G- and P-types were tested positive in the ApoH-ELISA in different dilutions. Compared to the qPCR results, the analysis showed high sensitivity, specificity and low cross-reactivity for the ApoH-ELISA, which was confirmed in receiver operating characteristics (ROC) analysis.

**Conclusions:**

In this study the development of a highly sensitive and specific capture ELISA was demonstrated by combining a poly-specific ApoH capture step with specific detection antibodies using group A rotaviruses as an example.

## Background

Apolipoprotein H (ApoH) is a human plasma protein able to bind poly-specifically to viruses. Coated onto magnetic beads or ELISA plates, it enables capture and concentration of viruses contained in critical diagnostic samples [1-3]. Common capture assays use specific poly- or monoclonal antibodies for capture and detection of viral antigens. These approaches have limitations since for each virus both a specific capture and a specific detection antibody are needed. In contrast, ApoH binds complete viruses and antigens poly-specifically and therefore offers the chance to capture and detect a broad range of different viruses within one diagnostic sample.

Rotaviruses are responsible for the majority of acute gastroenteritis infections occurring in young children world wide [4]. The genus *Rotavirus *belongs to the family of *Reoviridae *and, according to the antigenic and genetic variants of the VP6 region, can be divided into seven groups named A-G. The majority of human infections are caused by viruses of group A, which can further be differentiated into 23 G- and 32 P-types, respectively, by VP7 and VP4 antigens. The classification into sero- and genotype corresponds for G-types while it is inconsistent in the case of P-types [5,6].

Altogether, 10 G- and 11 P-types have been found in human group A rotavirus (RV-A) infections, while mostly types G1P[8], G2P[4], G3P[8], G4P[8] and G9P[8] have been circulating in Europe in recent years [7]. Additionally, in a few cases, reassortants of human strains, zoonotic strains and types such as G6, G8, G9, G10 and G12 were found [8]. The WHO recommends the use of enzyme immunoassays for the diagnosis of rotavirus infections [9].

This study was initiated to investigate the feasibility and suitability of a poly-specific virus capture ApoH-ELISA combined with subsequent sensitive and specific RV-A detection. The ApoH-ELISA was compared to a commonly used quantitative real-time PCR (qPCR), to correctly discriminate positive from negative samples by the presence of genome equivalents (GE) and to avoid misclassification by false positive or negative results using other EIAs. The usefulness of this new method was assessed in order to show a general feasibility for the detection of common and rarely circulating RV-A directly from stool samples after ApoH-mediated binding to the cavity of a microtitre plate and specific detection by antibodies. The intention of this study was to demonstrate the proof of principle of a poly-specific capture step with subsequent highly specific and sensitive detection of a pathogen and to analyse the potential of this method for further approaches.

## Results

### Suitability testing

In the first pre-testing steps it was shown that RV-A particles were bound to an ApoH-coated ELISA plate as shown by subsequent RV-A-specific qPCR. A loss of viral nucleic acid after the binding step to ApoH-ELISA plates was indicated by higher values (2 C_T_) in the qPCR assay compared to directly prepared samples without ApoH-ELISA incubation.

### Detection limit

The detection limit of this ApoH-ELISA assay was estimated by using dilutions of virus stocks obtained by cell culture with known virus load of infectious particles (TCID_50/ml_). Samples containing approximately 1,000 infectious units (TCID_50/ml_) were reactive in the ApoH-ELISA (Table 1, nos. 33-39). The best performance was seen when samples were diluted in NaOAc-buffer (data not shown).

**Table 1:** Results of qPCR and ApoH-ELISA of samples and controls (undiluted, 1:10 or 1:100 dilution)

**No**.	Sample ID	Genotype	Undiluted sample		1:10 dilution in NaOAc-buffer (50 mM)		1:100 dilution in NaOAc-buffer (50 mM)	
			direct (qPCR) GE	ELISA OD	direct (qPCR) GE	ELISA 1:10 OD	direct (qPCR) GE	ELISA OD
1	09g1014	G1P[8]	8.9E+06	2.5224	2.4E+05	3.0000	4.4E+04	3.0000
2	09g429	G1P[8]	7.8E+06	3.0000	1.7E+07	2.2098	2.1E+06	1.2997
3	09g454	G1P[8]	6.5E+06	3.0000	8.6E+05	1.5300	1.0E+05	0.3800
4	09g517	G2P[4]	5.4E+05	3.0000	6.2E+04	3.0000	5.3E+03	1.6400
5	09g991	G2P[4]	8.8E+05	0.6528	1.6E+06	1.8972	7.7E+05	1.1210
6	09g928	G3P[8]	2.4E+08	2.1567	2.9E+07	1.7056	3.6E+06	0.3692
7	09g449	G4P[8]	3.0E+05	3.0000	5.3E+04	1.5100	9.7E+03	0.3900
8	09g451	G4P[8]	1.4E+06	1.0200	8.9E+04	0.7900	1.2E+04	0.2100
9	09g452	G4P[8]	3.5E+08	3.0000	6.7E+07	1.9517	4.3E+06	0.7855
10	09g453	G4P[8]	4.3E+08	3.0000	1.1E+08	1.4608	1.9E+07	0.6903
11	09g1022	G8P(4)	7.2E+07	1.1735	9.0E+06	0.9167	6.7E+05	0.3529
12	09g962	G9P[8]	1.2E+03	3.0000	310	3.0000	20	3.0000
13	09g103	G9P[8]	1.9E+08	1.3311	9.0E+07	1.8673	3.9E+04	2.3071
14	09g410	G9P[8]	3.8E+05	0.2800	5.2E+04	0.1200	4.2E+03	0.0400
15	09g1028	G12P[6]	1.1E+08	2.3188	5.6E+06	1.4600	6.6E+05	0.3582
16	RoA cc1	WA	7.6E+06	2.6300	9.8E+05	2.6900	1.5E+05	2.4100
17	RoA cc2	WA	2.3E+07	2.7957	2.3E+06	2.2964	2.4E+05	0.5170
18	RoA cc3	WA	1.8E+07	2.8673	1.9E+06	2.2590	1.8E+05	0.4448
19	HAV_1	n.s.	4	0.4887	n.a.	0.3276	n.a.	0.1622
20	HAV_2	n.s.	16	0.4200	50	0.2200	12	0.0900
21	Human2	n.a.	n.a.	0.2356	n.a.	0.3802	n.a.	0.1210
22	Human3	n.a.	n.a.	0.0981	n.a.	0.2047	n.a.	0.0666
23	Wild boar	n.a.	n.a.	0.0393	n.a.	0.0384	n.a.	0.0367
24	Rat	n.a.	n.a.	0.0903	n.a.	0.1205	n.a.	0.0189
25	Mouse	n.a.	n.a.	0.0443	n.a.	0.0304	n.a.	0.0229
26	Pig_1	n.a.	n.a.	0.4158	n.a.	0.1632	n.a.	0.0573
27	Pig_2	G4P[8]	135	0.8721	2	0.4551	n.a.	0.1841
28	FCS	n.a.	n.a.	0.0203	n.a.	0.0189	n.a.	0.0228
29	NaOAc1	n.a.	n.a.	0.0358	n.a.	0.0340	n.a.	0.0354
30	NaOAc2	n.a.	n.a.	0.0821	n.a.	0.0582	n.a.	0.0658
31	NaOAc3	n.a.	n.a.	0.1896	n.a.	0.1233	n.a.	0.0700
32	NaOAc4	n.a.	n.a.	0.0217	n.a.	0.0176	n.a.	0.0001
33	CC_dil-2	WA	10,000 IU	1.6464	-	-	-	-
34	CC_dil-3	WA	1,000 IU	0.3173	-	-	-	-
35	CC_dil-4	WA	100 IU	0.0550	-	-	-	-
36	CC_dil-5	WA	10 IU	0.0399	-	-	-	-
37	CC_dil-6	WA	1 IU	0.0297	-	-	-	-
38	CC_dil-7	WA	0.1 IU	0.0286	-	-	-	-
39	CC_dil-8	WA	0.01 IU	0.0315	-	-	-	-

Abbreviations: IU: infectious units TCID_50/ml _measured in cell culture assay; GE: genome equivalents per reaction; OD: optical density at 450/620 nm; n.a.: no amplification; n.s.: no sequence result obtained; -: not done; CC_dil: dilution of cell culture virus stock

### ApoH-ELISA and qPCR analysis

This study was initiated together with the German Consultant Laboratory for Rotaviruses, using stored left-over samples from routine diagnostic which were pre-tested in commonly used EIAs and genotyped by PCR. The selection criteria for the stool samples were GE and genotype. Therefore, samples were included containing high and low GE of the most prevalent human strains circulating in Europe in recent years (G1P[8], G2P[4], G3P[8], G4P[8] and G9P[8]) as well as variants with low prevalence, G8P[4] and G12P[6], representing potentially zoonotic strains (Table 1). These samples were tested in this study in ApoH-ELISA and qPCR in parallel. Samples were classified into positive or negative based on the presence of GE as analysed with a specific and sensitive qPCR assay to avoid potential misclassification (false negative or positive) by using other EIAs.

The stool samples were tested in three different dilutions (undiluted, 1:10 and 1:100) to investigate the influence of inhibiting factors potentially present in those samples that might affect the results of the ELISA. Three of the 15 samples (Table 1; nos 1, 5, 13) showed increasing OD values in the ApoH-ELISA for dilutions, indicating inhibiting factors in the undiluted sample. All stool samples previously tested positive in PCR and EIA assays were confirmed positive in the specific qPCR. All stool samples from the diagnostic pool were reactive in the new ApoH-ELISA with OD values above 0.5 except for sample no. 14. This sample showed low values at or below the threshold in all three dilutions, although the GE in the qPCR were comparable to other samples (e.g. no. 4), which resulted in high OD values in the ApoH-ELISA. Two samples were highly reactive with values above 3 even in the 1:100 dilutions (nos. 1 and 12).

A broad range of different stool samples with unknown viral status from human adults and infants was included as control. Stool samples from different animals as well as buffers and FCS were tested for cross-reactivity. Sample no. 19 unexpectedly turned out to be reactive in the ApoH-ELISA while qPCR showed very low GE. A similar result was obtained when the original source sample (Table 1; no. 20) was tested again. The sample was from a hospitalized Hepatitis A Virus-positive patient with diarrhoea. Furthermore stool sample no. 27 taken from a pig which was stalled for an animal experiment was reactive in the ApoH-ELISA and positive in the qPCR assays with 135 GE. The GP-typing of the isolate showed a human G4P[8] origin. Sample no. 26 was negative in qPCR with relatively high OD values in the ApoH-ELISA. This sample originated from a second pig stalled close to the pig from which sample no. 27 originated, and it can be speculated that in this case viral antigen was present in the stool after oral up-take of contaminated faeces, without subsequent infection of the pig. Further control samples of different species (mouse, rat, etc.) and buffers (FCS, PBS) did not show any unspecific signals in the ApoH-ELISA in the absence of viral genomes. Positive controls from rotavirus cell culture supernatant showed corresponding values in both assays.

### Receiver operating characteristic (ROC) analysis

**Figure 1:**
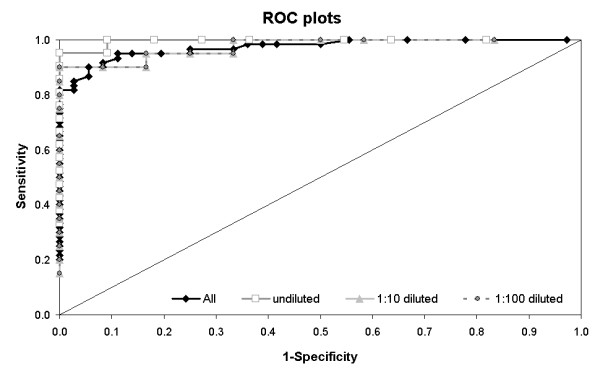
**ROC plot of the results for the detection of RV-A using the new ApoH-ELISA in comparison to the qPCR**. The figure contains the overlay of all results of the ROC (receiver operating characteristics) analysis for all samples (overall) and for each dilution step (undiluted, 1:10, 1:100) separately with the according area under the ROC curve (AUC). The results of the qPCR analysis of these samples were used as "gold standard" reference. The ROC plots the true positive rate (sensitivity) against the false positive rate (1-specificity). The diagonal indicates no discriminatory power. The plots of the ROC curve for the samples 1:10 and 1:100 dilution were superimposed on each other.

The estimation of the area under the ROC-curve (AUC) is a general test for diagnostic accuracy, and high values close to 1 indicate a highly accurate test [10]. The results of the qPCR analysis were set as the reference for the comparison of the ApoH-ELISA results. The ROC-analysis showed very high and comparable AUC-values exceeding 0.95, using either the results of each dilution step separately or all results together as one matrix (Figure 1, Table 2). The cut-off points of the method, adjusted to high specificity and high sensitivity, showed relatively low extinction values below 0.5 OD for the ApoH-ELISA. Also the analysis using the TG-ROC Excel sheet [11] gave high theta0 values close to 1 with a narrow intermediate range (IR) for the undiluted sample calculation.

**Table 2:** Results of the ROC analysis for three separate dilution steps and the combined data set

	AUC	Cutpoint	Sensitivity	Specificity	Correctly classified	LR+	LR-	theta0	d0 (range)	IR	upper limit (range)	lower limit (range)	VRP
**Undiluted**	0.9957	≥ 0.42	95.24%	100.00%	96.88%		0.0476	0.931	0.360 (0.283, 0.653)	0.000	0.416 (0.19, 0.416)	0.424 (0.283, 0.489)	1.003
**1:10 diluted**	0.9667	≥ 0.4551	90.00%	100.00%	93.75%		0.1	0.908	0.352 (3, 3)	0.000	0.328 (0.205, 0.328)	2.605 (3, 1.528)	1.764
**1:100 diluted**	0.9595	≥ 0.21	89.47%	100.00%	93.75%		0.1053	0.909	0.174 (3, 1.642)	0.000	0.162 (0.067, 0.162)	3.000 (3, 0.383)	1.946
**Overall**	0.9708	≥ 0.2122	95.00%	88.89%	92.71%	85.500	0.0562	0.917	0.264 (0.283, 0.416)	0.000	0.236 (0.09, 0.416)	0.571 (0.283, 1.017)	1.112

A non-parametric analysis was performed;

Abbreviations: Overall: all dilution steps were analysed together; AUC: area under the ROC-curve; LR+/LR-: Positive and negative likelihood ration; theta0/d0: co-ordinates at cut-off point of equivalence with specificity = sensitivity = theta0; IR: intermediate range, VRP: valid range proportion

### Analysis of dependency genome equivalents vs. OD values

The analysis of dependency between GE and OD extinction values was run using Spearman's rank test [12]. One test used only samples that had been tested positive in qPCR (qPCR = 1, n = 60) for comparison with the ELISA results. In the prediction test including only qPCR-positive samples Spearman's rho was 0.3113 (p = 0.016). Another test included all samples (n = 96) for the Spearman test, Spearman's rho was 0.7746 (p < 0.0001). This indicates that a correlation between GE and resulting OD in the ApoH-ELISA can be assumed, with a stronger correlation when using the whole data set also including negative samples.

## Discussion

The detection of different RV-A genotypes showed reliable results and good performance when using the newly established ApoH-ELISA. Even samples with very low GE in qPCR analysis were reactive. This might be due to a possibly higher amount of viral antigen in some stool samples compared to viral RNA. Since the time of sampling in the course of infection is unknown, no correlation could be drawn between antigen level or viral genomes and stage of infection. Surprisingly, one pig sample turned out to be positive for a human RV-A isolate. The fact that this was one of the pigs that were held for animal experiments and had daily contact to humans points to a possible human-to-pig infection route. Analysing the results of the three dilution steps per sample subjected to the ApoH-ELISA, all undiluted samples gave good results. However, increasing extinction values with increasing dilution steps in three samples indicated the presence of inhibiting factors that did not prevent detection of the infection but might result in underestimating the amount of antigen in the stool sample if applied undiluted only. A predictable correlation was shown between GE and OD values in the ELISA. This correlation was stronger when all samples, qPCR positive and negative, were included instead of analysing only qPCR positive samples.

Because it is common practice to use stool samples for the diagnosis of enteral viruses like rotavirus, we did not use any other material for the testing of the ApoH-ELISA, except cell culture supernatant during the prior establishment of the assay. The ApoH-ELISA showed a good sensitivity and specificity for the detection of common, rare and potentially zoonotic isolates of rotavirus, but the low number of stool samples used in this study is a limitation. As discussed by Philips *et al. *[13], ELISA reactivity seems to be a good correlate of disease in RV-A infection, which is in line with the protocol of the WHO for surveillance of rotavirus-associated gastroenteritis [9]. Because of the initial poly-specific capture step followed by antibody-dependent specific detection, this method could then be evaluated in future studies for the suitability in a differential diagnosis set-up for the simultaneous detection of other pathogens causing similar symptoms using just one capture ELISA plate. The parallel application of pathogen- or genotype-specific antibodies would allow then at the same time detection and discrimination on one plate and in one step. The aim of this study was to demonstrate the proof of principle and feasibility of the method. Therefore the rotavirus ApoH-ELISA requires an evaluation and validation with a quantitatively broader spectrum of clinically relevant stool samples before it can be used in routine diagnostics of rotaviruses. It will be necessary to analyse a higher number of samples with clinical data and it might be valuable to adopt other antibodies (mono- or polyclonal) for pathogen detection to further improve performance of the assay. It will also be worth to carefully evaluate the potential of multiplexing in one plate for simultaneous detection of different pathogens. Additionally the ApoH-ELISA will have to be compared to the "gold standard" EIAs (although the samples were all previously tested positive in standard EIAs used for routine diagnostic). Using rotavirus as a model, this study demonstrates the suitability of a new method for the specific and sensitive detection of viruses directly from stool samples by a poly-specific binding of antigen or complete virus to an ApoH-coated ELISA plate. The findings of the ApoH-ELISA are in accordance with a commonly used qPCR test system.

## Conclusion

The application of ApoH-ELISA was shown to be practicable for the diagnostics of RV-A in stool samples, providing results comparable to a commonly used qPCR analysis.

## Methods

### Test samples

A total of 15 human rotavirus-positive stool samples left over from routine diagnostic and obtained from the German Consultant Laboratory for Rotaviruses, were included (Table 1; nos. 1-15). These stool samples were pre-tested in commercially used EIAs and by broad-spectrum PCRs. The samples were additionally genotyped using a multiplex PCR for G- and P-type differentiation [14,15]. Altogether 15 relevant isolates of different genotypes and with different viral loads were chosen. The analysed samples belonged to the most common human viruses circulating in Europe during recent years and were typed as G1P[8], G2P[4], G3P[8], G4P[8] and G9P[8]. Additionally, G8P[4] and G12P[6] strains representing potentially zoonotic isolates were included. Different human stool samples with unknown viral status for rotavirus from adults and infants (Table 1; nos. 19-22) were also included in the analysis.

Furthermore, stool samples from other animal species (mouse, rat, pig, wild boar) as well as buffer and foetal calf serum (FCS, Biochrom, Berlin, Germany) served as controls to test potential cross-reactivity and unspecific background signals.

### ApoH-ELISA

A volume of approximately 100 μl/mg of each stool sample, virus supernatant or control sample was resuspended in 1 mL of NaOAc-buffer (50 mM). Solid particles were removed by centrifugation at 11,000 rpm for 2 min (Centrifuge 5417R, Eppendorf, Hamburg, Germany). The supernatant was removed and used undiluted, 1:10 and 1:100 diluted in NaOAc-buffer (50 mM). Altogether three samples (undiluted, 1:10, 1:100) of each original sample were used in the final analysis. One aliquot of each suspension (undiluted, 1:10, 1:100) was used for ApoH-ELISA detection, another for direct preparation and measurement of GE in a RV-A-specific qPCR.

For ApoH-ELISA analysis 100 μl of the suspension were given directly onto the ApoH-ELISA plate. The plate was then centrifuged for enhanced virus binding at 2,500 rpm for 15 min (Centrifuge Biofuge Stratos, Heraeus, Thermo Fisher Scientific, Waltham, MA, USA) before incubation of the plate for 1 h at 37°C in a CO_2 _cell incubator (CB150, Binder, Tuttlingen, Germany) in a humid atmosphere. The supernatant was then removed from the plate and discarded. The wells were washed at least three times with 200 μl washing buffer PBST (PBS with 0.1% Tween20 [Sigma-Aldrich, Hamburg, Germany]). A blocking step with 200 μl blocking buffer (PBST with 2% skimmed milk powder, Sigma-Aldrich) for 45 min at room temperature was introduced to reduce unspecific signals. The supernatant was then discarded and another washing step was performed using PBST. A polyclonal rabbit anti-rotavirus (human) antibody (DAKOCytomation, Hamburg, Germany) diluted 1:100 in blocking buffer was used in a volume of 100 μl as first antibody and was incubated on the plate for 1 h at room temperature. The antibody was then removed. After washing, 100 μl of the secondary antibody were applied for 1 h at room temperature (goat anti-rabbit POD H&L IgG 1:5000 diluted in blocking buffer, Dianova, Hamburg, Germany) followed by an additional washing step. Finally 100 μl of TMB (3,3',5,5'-Tetramethylbenzidine) diluted in substrate buffer (Bio-Rad, Munich, Germany) were added to each well of the plate. The reaction was stopped with 1 N H_2_SO_4 _after incubation for 20 min at room temperature in the dark. The plate was analysed in a plate reader (TECAN Infinite F200, Männedorf, Switzerland) at 450/620 nm and extinction values were calculated using Magellan™ Software (TECAN).

### Quantitative real-time PCR

One aliquot of the respective dilution for each sample which was tested in the ApoH-ELISA was analysed in parallel by qPCR to determine the GE of RV-A. A volume of 100 μl of each sample was resuspended directly in 400 μl AVL lysis buffer (RNeasy viral RNA kit, Qiagen, Hilden, Germany) and incubated for 10 min at room temperature followed by RNA extraction according to the manufacturer's recommendations. Subsequently, a volume of 20.6 μl of the eluted RNA was reverse transcribed using the TaqMan^® ^Reverse Transcription Reagents following the supplier's protocol (Applied Biosystems, Foster City, USA). RNA was incubated with 2 μl primer mix of random hexamers (R6)/specific RoA_25/26 primers (RoA_25 gCT TTT AAA AgT TCT gTT CCg Ag; RoA_26 CTC AAT gTg TAR TTg Agg TCg, TIB Molbiol, Berlin, Germany) for 30 sec at 93°C. The sample was snap-cooled on ice to 4°C before adding the reverse transcription mix containing 4 μl 10xbuffer, 8.8 μl MgCl_2 _(25 mM), 3.2 μl dNTP (25 mM), 0.8 μl RNase inhibitor and 0.6 μl reverse transcriptase. The reaction was run in a thermocycler (Mastercycler epgradient, Eppendorf) for 60 min at 42°C followed by 15 min at 72°C before cooling to 4°C and storage at -20°C.

The GE of rotavirus were determined by a qPCR assay, using 5' nuclease probes (TaqMan^® ^probes) with primer combination RoA_25/26 and probe TM3 (RoA_TM3 probe YAK-CTT ITC CAT CTT TCC gCA CgC gCT--DB, TIB Molbiol), which was designed to detect all human and animal RV-A by binding within a conserved region of the NSP4 gene.

The Platinum^® ^Taq DNA polymerase kit, MgCl_2 _and dNTPs by Invitrogen (Carlsbad, CA, USA) together with water (Molecular Biology Grade Eppendorf) were applied in this assay. qPCR reactions were performed in a final volume of 25 μl with 2.5 μl sample, 10xbuffer, 4 mM of MgCl_2_, dNTP 0.2 mM each, 0.3 μM of each primer, 0.1 μM of probe, ROX 0.1 μM and Platinum^® ^Taq 0.5 IU. General reaction conditions for the real-time assay were 95°C for 15 min and 45 cycles with 95°C for 15 sec and 60°C for 35 sec. Reactions were run in an ABI GeneAmp^® ^7500 Detection System (Applied Biosystems). A plasmid pRoA containing the corresponding region of 75 nt from the isolate RV-A WA (ATCC VR-2018^®^) was established. Plasmid DNA was purified, concentration was determined and copy numbers were calculated. The plasmid was 10-fold serially diluted in water containing λ-DNA (1 ng/μl) from 10^6 ^to 10 copies and used as positive control as well as for quantification of viral genomes. For qPCR each sample was analysed in duplicate. Genome equivalents were determined using a standard curve; the mean and standard deviation were calculated. The detection limit was calculated to be 10 cDNA copies per reaction.

As internal sample control a c-myc qPCR assay with primer combination c-myc_F/R/TM (c-myc F: gCC AgA ggA ggA ACg AgC T; c-myc R: ggg CCT TTT CAT TgT TTT CCA; c-myc TM FAM-TgC CCT gCg TgA CCA gAT CC-BBQ; TIB Molbiol) and a quantified plasmid containing the target sequence of 83 nt were applied for all stool samples. The qPCR reaction conditions corresponded to the RV-A assay.

### ApoH-ELISA - suitability testing

In advance, tests to analyse the general binding of rotavirus particles to ApoH-coated ELISA plates and optimization steps using different dilution buffers were performed. For the establishment of the method cell culture grown virus stock RV-A strain WA with a titre of 1.3 × 10^7 ^IU_/mL _TCID_50 _[16,17] was used. The virus suspension was 10-fold serially diluted from 10^-1 ^to 10^-8 ^in NaOAc-buffer (50 mM). One aliquot of each dilution step was used for the ApoH-ELISA assay, and another aliquot was analysed directly by qPCR for determination of GE in the sample. First, aliquots of the different sample dilutions were incubated on the ApoH-ELISA plate for 1 h at 37°C. Supernatant was removed, the plate was washed twice with 200 μl PBST and lysis of bound virus particles was performed directly on the plate using AVL lysis buffer (RNeasy viral RNA kit, Qiagen). After 10 min of incubation at room temperature samples underwent RNA extraction and cDNA synthesis and were stored for further analysis as described before. A corresponding aliquot was used directly for RNA extraction, cDNA synthesis and qPCR analysis.

Subsequently, analysis was performed using diluted cell culture supernatant and ApoH-ELISA to estimate the detection limit of the ELISA assay by using defined samples with known virus titre. Therefore the threshold of the OD values of the ApoH-ELISA can be correlated to the detection limit of infectious units (TCID_50/ml_) in the sample. Cell cultured virus was diluted 10-fold from 10^-1 ^to 10^-8 ^either in PBS, NaOAc- (50 mM) or Tris-buffer.

### Receiver operating characteristic analysis

Receiver operating characteristic (ROC) analysis was used to determine a cut-off value (OD) for the newly established ApoH-ELISA [10,18]. The commonly applied qPCR was used as reference test to indicate presence or absence of virus in the respective sample and to rule out misclassification of samples due to false-negative or -positive results by other EIA test. With the Youden Index (sensitivity+specificity-1 [19]) the optimal cut-off for each dilution step separately and for all samples as a whole was identified. The ROC analysis was performed using either the whole data set or the results of each dilution step separately. The area under the curve (AUC) was estimated for each of the three dilution steps separately and for the whole data set. The result of each analysis was compared to determine a difference in performance by using dilution steps. Positive and negative likelihood ratios (LR+ and LR-) were measured and the analysis was performed using STATA11 (StataCorp LP, College Station, TX, USA) and TG-ROC Excel-sheet [11].

### Statistical analysis

Spearman test [12] was run using STATA11 (StataCorp LP) to analyze the correlation between GE in qPCR and corresponding OD values in ApoH-ELISA.

## Competing interests

IS is chief executive officer (CEO) of ApoH-Technologies SA, FV is a co-founder and scientific advisor of ApoH-Technologies SA. All other authors declare no competing interests.

## Authors' contributions

CA: conception, design, analysis, interpretation of data and manuscript draft; MK: design, analysis, manuscript draft; MH: design, reference material, analysis support, manuscript revision; AM: pretesting of samples, genotyping assay, manuscript revision; IS: conception, ApoH-ELISA set-up, delivery of ELISA-plates, manuscript revision; FV: conception, ApoH-ELISA set-up, delivery of ELISA-plates, manuscript revision; HE: study design, interpretation of data, final manuscript approval All authors have read and approved the final manuscript.
